# Stem cell-derived polarized hepatocytes

**DOI:** 10.1038/s41467-020-15337-2

**Published:** 2020-04-03

**Authors:** Viet Loan Dao Thi, Xianfang Wu, Rachel L. Belote, Ursula Andreo, Constantin N. Takacs, Joseph P. Fernandez, Luis Andre Vale-Silva, Sarah Prallet, Charlotte C. Decker, Rebecca M. Fu, Bingqian Qu, Kunihiro Uryu, Henrik Molina, Mohsan Saeed, Eike Steinmann, Stephan Urban, Roshni R. Singaraja, William M. Schneider, Sanford M. Simon, Charles M. Rice

**Affiliations:** 1 Laboratory of Virology and Infectious Diseases, The Rockefeller University, New York, NY USA; 2 Schaller Research Group at Department of Infectious Diseases and Virology, Heidelberg University Hospital, Cluster of Excellence CellNetworks, Heidelberg, Germany; 3 Laboratory of Cellular Biophysics, The Rockefeller University, New York, NY USA; 4 Proteomics Resource Center, The Rockefeller University, New York, NY USA; 5 Department of Biology, New York University, New York, NY USA; 6 Department of Infectious Diseases and Virology, Heidelberg University Hospital, Cluster of Excellence CellNetworks, Heidelberg, Germany; 7 German Center for Infection Research (DZIF), Partner Site Heidelberg, TTU Hepatitis, Germany; 8 Electron Microscopy Resource Center, The Rockefeller University, New York, NY USA; 9 Department of Molecular and Medical Virology, Ruhr-University Bochum, Bochum, Germany; 10 A*STAR (Agency for Science, Technology and Research) Institute and Yong Loo Lin School of Medicine, National University of Singapore, Singapore, Singapore; 11 Present Address: Huntsman Cancer Institute, University of Utah, Salt Lake City, UT 84105 USA; 12 Present Address: Department of Molecular, Cellular and Developmental Biology, Microbial Sciences Institute, Yale University, West Haven, CT 06516 USA; 13 Present Address: Department of Bioinformatics and Functional Genomics, Biomedical Computer Vision Group, BIOQUANT, IPMB, University of Heidelberg, Heidelberg, Germany

**Keywords:** Stem-cell biotechnology, Cell polarity, Virology, Stem-cell differentiation

## Abstract

Human stem cell-derived hepatocyte-like cells (HLCs) offer an attractive platform to study liver biology. Despite their numerous advantages, HLCs lack critical in vivo characteristics, including cell polarity. Here, we report a stem cell differentiation protocol that uses transwell filters to generate columnar polarized HLCs with clearly defined basolateral and apical membranes separated by tight junctions. We show that polarized HLCs secrete cargo directionally: Albumin, urea, and lipoproteins are secreted basolaterally, whereas bile acids are secreted apically. Further, we show that enterically transmitted hepatitis E virus (HEV) progeny particles are secreted basolaterally as quasi-enveloped particles and apically as naked virions, recapitulating essential steps of the natural infectious cycle in vivo. We also provide proof-of-concept that polarized HLCs can be used for pharmacokinetic and drug-drug interaction studies. This novel system provides a powerful tool to study hepatocyte biology, disease mechanisms, genetic variation, and drug metabolism in a more physiologically relevant setting.

## Abstract (web summary)

To model hepatocyte function accurately in vitro, it is necessary to generate and maintain a polarized epithelium. Here, the authors describe a protocol to generate polarized human pluripotent stem cell-derived hepatocyte-like cells (HLCs) to model enteric virus production and drug secretion in vitro.

## Introduction

A major function of the liver is to filter blood from the digestive tract before it passes through the body. This task is performed by hepatocytes, which filter and process blood nutrients, metabolites, hormones, drugs, and other compounds for storage and excretion. Hepatocytes thus form a crucial cell layer engaged in two counter-current flow systems, which, on the one hand, involve uptake, processing, and secretion of sinusoidal blood components, and on the other hand, synthesis and secretion of bile^1^.

**Fig. 1:**
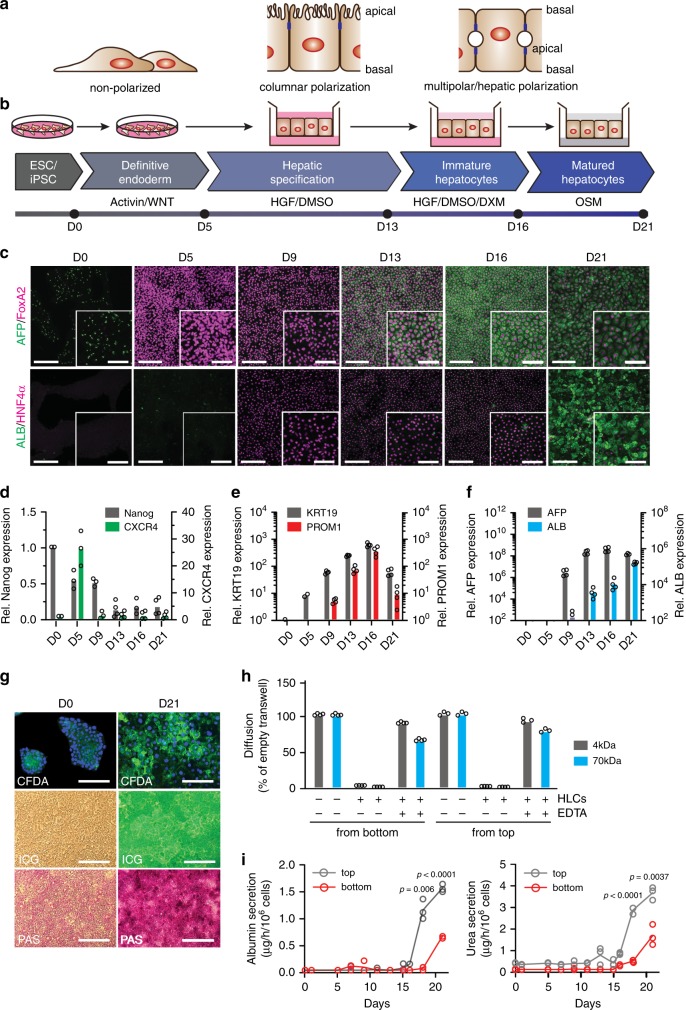
Stem cell-based differentiation on transwells to generate polarized hepatocyte-like cells (pol-HLCs). **a** Schematic depicting the cellular organization of non-polarized, columnar polarized, and hepatocyte/multi-polarized cells. Blue boxes are tight junctions separating apical and basal membranes. **b** Stem cell differentiation protocol on transwells to generate pol-HLCs. **c** Representative immunofluorescence images of hESCs (day 0), definitive endoderm (day 5), hepatic progenitor (day 9 and 13), immature HLCs (day 16), and pol-HLCs (day 21). Cells were stained for immature hepatocyte markers AFP (green) and FoxA2 (magenta) or for mature hepatocyte markers ALB (green) and HNF4α (magenta). Scale bars = 500 μm/250 μm. **d**–**f** RT-qPCR analysis of the indicated genes along the pol-HLC differentiation protocol relative to D0 (*n* = biological replicates). **g** Pol-HLCs metabolize carboxyfluorescein diacetate (CFDA) and indocyanine green (ICG), and store glycogen as evidenced by periodic acid-schiff staining (PAS) staining. Scale bars = 250 μm **h** Paracellular permeability of polarized HLCs incubated O/N with 4 kDa FITC-dextran or 70 kDa RITC-dextran in the absence or presence of EDTA, plotted relative to diffusion across empty filters (*n* = biological replicates). **i** Rate of albumin (left panel) and urea (right panel) secretion into either the top or bottom compartment during pol-HLC differentiation (*n* = biological replicates). Statistical analysis was performed using a two-tailed unpaired *t* test with Bonferroni adjustment for multiple comparisons.

To mediate these functions, hepatocytes have a unique polarization with multiple basolateral membranes facing the sinusoids, and multiple apical membranes forming bile canaliculi (Fig. 1a, right panel). Within this peculiar structure, cell signaling, membrane trafficking, protein secretion, and bile transport are highly organized^2^. Although well described morphologically, little is known about the molecules that orchestrate polarization or their regulation, as a robust polarized system to study authentic hepatocyte function is lacking. Research tools are limited because few human hepatoma cell lines can be polarized^1^; and those that can be polarized are typically de-differentiated with altered proliferative, metabolic, immune, and apoptotic responses.

Although primary human hepatocytes (PHH) offer a better alternative, they are not extensively used due to their limited availability, high donor-to-donor variability, and limited usefulness for genetic manipulation. In addition, PHH often de-differentiate upon plating and lose their hepatic morphology and functions^1,3^. To overcome these limitations, PPH can be expanded in vivo in liver injury mouse models and subsequently plated for in vitro studies^4,5^. Yet, potential co-purified mouse cells may, depending of the assay applied, complicate the interpretation of results. Sandwich or micropattern PHH cultures with supporting stromal cells^3^ maintain hepatic polarization, but neither the biliary membrane nor the cargo excreted into the closed bile canaliculi are readily accessible (Fig. 1a, right panel). Furthermore, the extracellular matrix overlay hinders solute diffusion and complicates live cell imaging studies. Hepatocyte-based research would thus benefit from more reliable, physiologically relevant, and more experimentally tractable hepatocellular polarity systems.

For these reasons, human embryonic or induced pluripotent stem cell (hESC/iPSC)-derived hepatocyte-like cells (HLCs^6,7^) could offer an attractive option to fill this need for a polarized hepatocyte culture model. Here we report a novel stem cell-based differentiation protocol that generates columnar polarized HLCs. These polarized HLCs secrete cargo directionally and allow non-invasive sampling of both compartments. Albumin, urea, and lipoproteins are secreted basolaterally, with bile acids secreted apically. Enterically transmitted hepatitis E virus (HEV) progeny particles are secreted basolaterally as quasi-enveloped particles and apically as naked virions, recapitulating the natural history of infection in vivo. We also provide proof-of-concept that polarized HLCs offer an attractive platform to test and model drug–drug interaction studies.

## Results

### Generation of HLCs exhibiting columnar polarization

As described above, maintenance of cell polarity is essential for retaining PHH functions, yet, HLCs are conventionally differentiated in culture dishes under two-dimensional (2D) culture conditions, a process that is inefficient, variable, and yields less-polarized cells. The resulting HLCs are immature and resemble fetal rather than adult hepatocytes^6^. When HLCs are differentiated in spheroids^8,9^ or cultured in micropatterned co-cultures^10^ they better recapitulate hepatocyte functions. This suggests that three-dimensional (3D) architecture assists hepatocyte maturation.

Most epithelial cells, such as lung or intestinal cells, are columnar polarized (Fig. 1a). Some evidence exists that hepatocyte differentiation passes through a columnar intermediate in vivo^1^. Therefore, we adapted an existing stem cell-based HLC differentiation protocol^11^ to generate columnar polarized HLCs on transwell filters (Fig. 1b). In the past, transwell filters have been extensively used for columnar polarization (Fig. 1a) of a range of cancer epithelial cells. As the transwell filter is permeable, the configuration permits uptake and secretion of molecules by both, the basolateral and apical sides of the cell, allowing metabolic activities to occur in a more physiological fashion.

We first differentiated hESCs to definitive endoderm (DE) in culture dishes. By day 5, cells expressed lower levels of the pluripotency marker Nanog compared with hESC cells (Fig. 1d) and high levels of DE markers forkhead box protein A2 (FoxA2) (Fig. 1c) and C-X-C Motif Chemokine Receptor 4 (CXCR4) (Fig. 1d). To induce hepatic specification, we then seeded the DE cells on matrigel-coated transwell filters in serum-free, hepatocyte growth factor (HGF)-containing medium. Expression of the biliary markers keratin-19 (KRT19) and prominin-1 (PROM1) increased by day 9 (Fig. 1e) and plateaued by day 13, at which point the cells expressed nuclear hormone receptor HNF4α, an accepted marker of human hepatic progenitor (HepProg) cells^12^ (Fig. 1c). We further matured the HepProg cells by exposing them to basolateral medium without growth factors in the top compartment, and to complete medium supplemented with HGF and dexamethasone in the bottom compartment. This process yielded immature hepatocytes (ImHep), which by day 16 expressed high levels of the fetal liver marker alpha-fetoprotein (AFP) (Fig. 1c and f). ImHeps were further matured by exposing them to basic hepatocyte culture medium (HCM) in the top compartment and complete HCM medium supplemented with oncostatin-M in the bottom compartment. By day 21, this process yielded HLCs that expressed high levels of the adult hepatocyte marker albumin (ALB) (Fig. 1c and f). By counting ALB-positive cells, we determined that ~80% of the final cell population consisted of HLCs (Fig. 1c). Notably, HLCs underwent hepatic multipolar polarization and formed apparent bile canaliculi when grown on transwell filters with fully supplemented medium in both compartments and overlaid with matrigel throughout differentiation.

Having successfully generated HLCs on transwell filters, we next examined several characteristic hepatic functions. The cells stained positive with indocyanine green, a tricarbocyanine dye that is taken up by hepatocytes (Fig. 1g). The HLCs also demonstrated the ability to synthesize glycogen, as tested by periodic acid-Schiff staining. We further demonstrated that HLCs could metabolize non-fluorogenic carboxyfluorescein diacetate into fluorogenic carboxyfluorescein (Fig. 1g). Next, we showed that the cells formed a tight monolayer by measuring transepithelial electrical resistance (~400 Ω/cm^2^) and assessing diffusion of small fluorescent dextrans (4 kDa and 70 kDa), which could not be detected in the opposite transwell compartment in the presence of polarized HLCs (Fig. 1h). From day 16 of the differentiation protocol, HLCs secreted characteristic hepatocyte cargos such as ALB and urea (Fig. 1i). This coincided with positive ALB staining (Fig. 1c).

This protocol was generalizable to multiple stem cell lines, including hESC lines RUES2^13^ and HUES8-iCas9^14^ (Supplementary Fig. 4) as well as the iPSC line iPS.C3A^15^. Importantly, the majority of ALB and urea, normally secreted by native hepatocytes from their basolateral membrane into the bloodstream, was secreted into the bottom compartment (Fig. 1i). These results indicate that the cells were differentiated into metabolically functional polarized HLCs (pol-HLCs), with what appeared to be simple, columnar epithelial polarization (Fig. 1a). Of note, pol-HLCs continued to secrete ALB and urea for another 22 days and 17 days, respectively (Supplementary Fig. 1).

### Properties of transwell-polarized HLCs

**Fig. 2:**
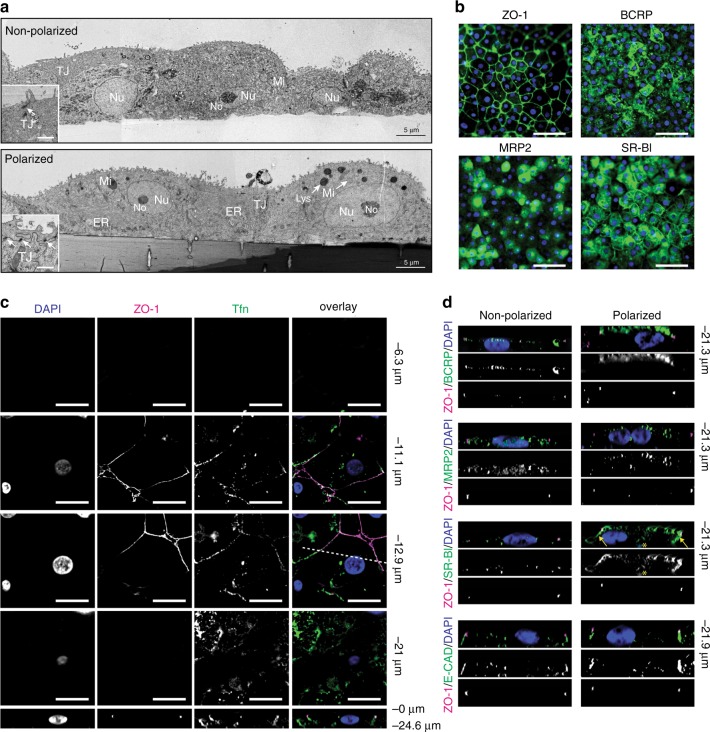
Structural polarization and organization of polarized HLCs. **a** Transmission electron microscopy, cross-sectional view of nonpol- and pol-HLCs. No, nucleolus; Nu, nucleus; Mi, mitochondria; ER, endoplasmic reticulum; Lys, lysosome; TJ, tight junctions. Scale bar in insets = 2 μm. **b** *xy* images of pol-HLCs stained for the tight-junction marker ZO-1 (green), breast cancer resistance protein (BCRP, green), multi-drug resistance protein 2 (MRP2, green), or scavenger receptor-B1 (SR-BI, green), and DAPI (blue). Scale bars = 200 μm **c** Transferrin-conjugate binding to pol-HLC. Pol-HLCs were incubated with 25 µg/mL Transferrin-594 (green) for 10 min at 37 °C prior to washing and staining with anti-ZO-1 (magenta). Bottom panels: *xz* images from cross sections indicated by the dashed line in the corresponding *xy*-images above. Scale bars = 15 μm. **d** Cross-sectional views (*xz*) of nonpol- and pol-HLCs stained for indicated marker. Yellow arrows = basolateral membrane of pol-HLCs. * = autofluorescence of transwell pore. Images are representative of three independent differentiations.

We then compared the cellular structure of pol-HLCs with conventionally differentiated HLCs on Matrigel-coated culture dishes. As shown in Fig. 2, conventional HLCs exhibited some level of cellular polarity with what appeared to be apical villi (Fig. 2a) and polarized distribution of some of the proteins analyzed (Fig. 2d). Yet, their degree of polarization is markedly less compared with pol-HLCs differentiated on transwells, as demonstrated in the following experiments. For simplicity, we refer to conventionally plated HLCs that have not been through our columnar polarization protocol as “nonpol-HLCs”.

Transmission electron microscopy revealed, that pol-HLCs were better organized, with a highly developed endoplasmic reticulum, clearly defined nuclei and mitochondria, and intact tight junctions (Fig. 2a). Therefore, the polarization on transwells likely helps HLCs maintain a healthy cellular structure.

We then examined the structural polarization of pol-HLCs by analyzing hepatic membrane proteins. We first confirmed their expression in pol-HLCs (Fig. 2b) and then analyzed their 3D distribution (Fig. 2d). According to their biological function, these proteins are expressed either on the apical or basolateral hepatocyte membrane. For example, the transferrin receptor is almost exclusively localized on the basolateral compartment of polarized epithelial cells^16^. Fluorescently conjugated human transferrin was added in equal amounts to both top and bottom transwell compartments to determine transferrin receptor binding. As shown in Fig. 2c, the transferrin-conjugate bound only to the membrane facing the bottom compartment, consistent with localization of the transferrin receptor on or near the basolateral membrane. We then compared immunofluorescent staining of other hepatocyte polarity markers on pol- and nonpol-HLCs (Fig. 2d). In pol-HLCs, the apical hepatic markers, breast cancer resistance protein (BCRP) and multi-drug resistance protein 2 (MRP2), were exclusively localized on the upper (apical) membrane, whereas the lipoprotein receptor scavenger receptor-B1 (SR-BI) was mainly localized on the bottom (basolateral) membrane (indicated by arrows in Fig. 2d). In contrast, nonpol-HLCs were flattened relative to pol-HLCs, and the marker distribution was less defined. Both pol- and nonpol- HLCs expressed the adherens junction protein E cadherin together with the tight-junction protein ZO-1 at cellular junctions (Fig. 2b–d), indicating that the cells developed proper cell–cell junctions in both configurations. Together, these results indicate that pol-HLCs acquire a simple, columnar polarization with an apical membrane facing the top transwell compartment and a basolateral membrane facing the bottom compartment.

### Directional secretion of hepatic cargo

A major function of hepatocytes is to regulate cholesterol levels in the body. Hepatocytes achieve this by synthesizing and secreting lipoproteins from their basolateral surface into the bloodstream for delivery to the periphery. They further remove excess cholesterol from the body by converting it into bile acids, which are then secreted from the hepatocyte’s apical compartment and eliminated as waste. To test if pol-HLCs could perform these activities, we performed RNA-Seq analysis to assess whether the relevant enzymes are expressed, then we measured the selectivity and directionality of lipoprotein and bile acid release.

**Fig. 3:**
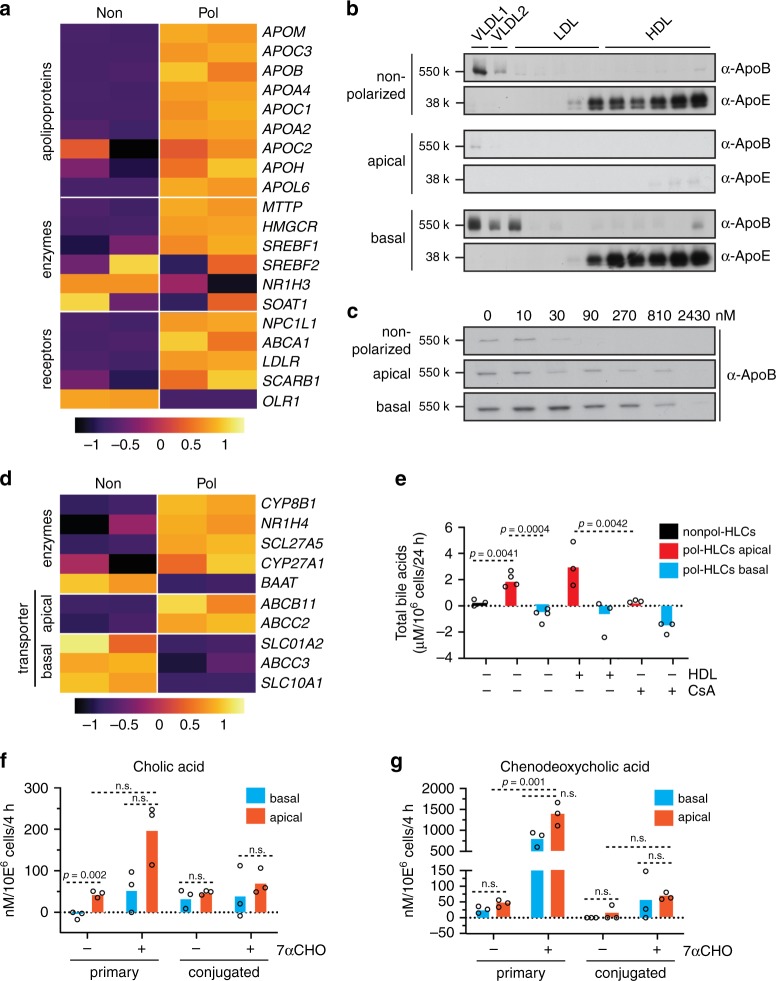
Vectorial hepatic cargo secretion from polarized HLCs. **a** Heatmap of *Z* score-normalized CPM values for enzyme and transporter genes involved in lipoprotein metabolism in nonpol- compared with pol-HLCs (*n* = biological replicates). **b** Density distribution of ApoB100 and ApoE secreted from nonpol- and pol-HLCs, labeled metabolically for 4 h with [^35^S] methionine/cysteine. Supernatants from labeled cells were subjected to density gradient centrifugation followed by ApoB100 or ApoE immunoprecipitation of each fraction, separation by SDS-PAGE, and detection by [^35^S] fluorography. Results are representative of three independent differentiations. **c** Nonpol- and pol-HLCs were treated with indicated concentrations of the MTP inhibitor Lomitapide, prior to metabolic labeling, ApoB100-immunoprecipitation and detection. Results are representative of three independent differentiations. **d** Heatmap for *Z* score-normalized CPM values of enzyme and transporter genes involved in bile acid metabolism in nonpol- (Non) compared with pol-HLCs (Pol) (*n* = biological replicates). **e** Total bile acid release from nonpol- and pol-HLCs treated for 24 h with 10 μg/ml high-density lipoprotein (HDL) or 10 μm cyclosporine A (CsA), as indicated (*n* = biological replicates). **f**–**g** LC-MS analysis of primary and conjugated cholic acid (**f**) and chenodeoxcycholic acid (**g**) released from pol-HLCs treated with DMSO or 10 μm 7α-hydroxylcholesterol (7α-CHO) for 4 h (*n* = biological replicates). Statistical analysis was performed using a two-tailed unpaired *t* test with Bonferroni adjustment for multiple comparisons.

RNA-Seq analysis revealed that the lipoprotein biosynthetic enzymes, apolipoproteins (Apo; key structural proteins of lipoproteins), and lipoprotein receptors were generally upregulated in pol-HLCs compared with nonpol-HLCs (Fig. 3a). We confirmed this trend by quantifying key genes by qRT-PCR analysis (Supplementary Fig. 2). We and others have previously shown that even nonpol-HLCs can synthesize and release ALB, ApoB100 and ApoE^17,18^. Here, we found that pol-HLCs released the majority of these cargos directionally from their basolateral membrane (Supplementary Figs. 3).

Hepatocytes also synthesize fatty acids, de novo, that are incorporated into triglycerides and exported as very-low-density lipoproteins (VLDL). Hepatoma cells typically do not make high levels of VLDL owing to a defect in their ability to fully lipidate ApoB100, the main non-exchangeable cargo protein of VLDL. As a result, hepatoma cells make low-density lipoprotein (LDL) rather than VLDL^19^. We analyzed newly secreted lipoprotein species from nonpol- and pol-HLCs by metabolically labeling the lipoproteins followed by separation via density gradient centrifugation. Both pol- and nonpol-HLCs fully lipidated ApoB (Fig. 3b). In agreement with a recent study^18^, this suggests that HLCs in general, unlike hepatoma cells, have a functional VLDL biosynthesis pathway. Importantly, pol-HLCs secreted higher levels of lipoproteins than nonpol-HLCs, and these lipoproteins were selectively secreted into the basolateral compartment (Fig. 3b). ApoB100 secretion decreased when the cells were treated, in the basolateral compartment, with the microsomal triglyceride transfer protein (MTTP) inhibitor Lomitapide (Fig. 3c). Higher concentrations of the MTTP-inhibitor were needed to block ApoB100 secretion by pol-HLCs compared with nonpol-HLCs, possibly due to their ability to eliminate drugs by secretion into the apical compartment.

Overexpression of human Apo-CIII in rat hepatocytes enhances VLDL production^20^. In the absence of a suitable culture system, it has not been possible to test the role of Apo-CIII in lipoprotein secretion from human hepatocytes. Given that HLCs can efficiently synthesize lapidated VLDL, we used the parental stem cell line HUES8-iCas9^14^ with inducible CRISPR-Cas9 to create Apo-CIII knockout cell clones (Supplementary Fig. 4a, b)^21^. Surprisingly, under lipid rich conditions, the absence of Apo-CIII in pol-HLCs had no observable effect on lipoprotein assembly or secretion (Supplementary Figs. 4c and d).

Next, we examined bile acid synthesis and release. RNA-Seq and Ingenuity pathway analysis showed an upregulation of genes involved in the farnesoid X receptor/retinoid X receptor and liver X receptor/-retinoid X receptor pathways in pol-HLCs compared with nonpol-HLCs (Fig. 3d). These pathways regulate lipid metabolism and catalyze cholesterol to bile acid metabolism^22^. Pol-HLCs expressed higher levels of major enzymes responsible for converting cholesterol to bile acid. Apical bile acid transporters, in contrast to basolateral transporters, were upregulated in pol-HLCs (Fig. 3d).

We then measured bile acid secretion from nonpol-HLCs as well as from the apical and basolateral sides of pol-HLCs (Fig. 3e). Nonpol-HLCs secreted bile acids at low levels. In contrast, pol-HLCs secreted high levels of bile acids into the apical compartment and only trace amounts into the basolateral compartment. Apical bile acid release could be stimulated by incubating cells with cholesterol in the form of high-density lipoprotein (HDL), and inhibited by treating cells with the cholestasis-causing drug cyclosporine A (Fig. 3e).

PHHs mainly secrete glycine or taurin conjugates of cholic acid (CA) and chenodeoxycholic acid (CDCA), whereas HepG2 hepatoma cells secrete mainly precursors and unconjugated bile acids^23^. HLCs, probably owing to their immature nature, were reported to secrete unconjugated CA and CDCA^24^. We therefore used liquid chromatography-mass spectrometry (LC-MS) to profile released bile acid species. Pol-HLCs synthesized and released the two primary bile acids, CA and CDCA, mainly from the apical side into the top compartment. Of note, unlike nonpol-HLCs^24^, pol-HLCs secreted both bile acid species and their conjugates at a similar ratio (Fig. 3f and g). Incubation with the cholesterol-derived bile acid precursor 7α-hydroxylcholesterol^25^ (7α-CHO) increased CA and CDCA synthesis and release from pol-HLCs (Fig. 3f and g). Although the majority of CA and CDCA was still released from the apical side, we also observed some level of basal secretion, suggesting that treatment of pol-HLCs with high doses of 7α-CHO induced excessive bile acid synthesis.

These results indicate that pol-HLCs exhibit improved lipoprotein synthesis, cholesterol to bile acid conversion, and bile acid release compared with nonpol-HLCs. In addition, the observed directional cargo release further confirmed the functional polarity of pol-HLCs.

### Polarized enteric virus release

Hepatitis A and E viruses (HAV, HEV) exploit unique properties of hepatocyte polarization that standard tissue culture systems fail to support. In a natural infection, these viruses enter polarized hepatocytes through the basolateral membrane, replicate, and produce progeny viruses. It is thought that progeny viruses then egress from the hepatic basolateral membrane into the bloodstream to spread within the host, and from the apical membrane into the bile and feces to infect new hosts (reviewed in refs. ^26,27^). Owing to the absence of viral glycoproteins, HAV and HEV are classified as non-enveloped viruses. Recently, it has been found that both viruses acquire a host cell-derived exosomal membrane-like “quasi-envelope” by budding into multivesicular bodies^28,29^. HAV and HEV particles circulating in the patient blood are quasi-enveloped, whereas those found in the feces are non-enveloped and highly infectious^28,29^. To date, experimental evidence that progeny HAV and HEV particles differentially bud from the hepatocytes’ apical and basolateral membranes is lacking.

**Fig. 4:**
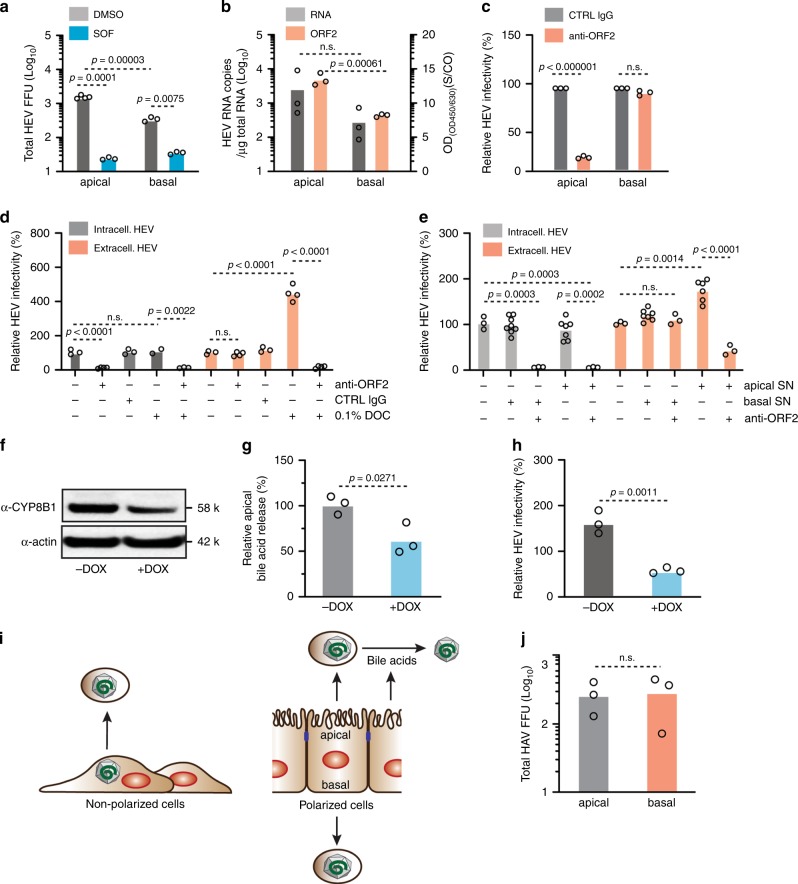
Modeling polarized infection of hepatitis A and E viruses. **a** Newly secreted focus-forming infectious particles (FFU) from HEV infected pol-HLCs released in either apical or basolateral compartment were titered on hepatoma cells. Pol-HLCs were treated with DMSO or 10 μm of the HEV replication inhibitor Sofosbuvir (*n* = biological replicates). **b** HEV particles released from HEV infected pol-HLCs 7 d post infection were also measured by qRT-PCR quantification of viral RNA copies or ORF2 capsid ELISA (*n* = biological replicates). **c** Newly secreted infectious HEV particles from infected pol-HLCs were treated with 1:100 anti-HEV capsid ORF2 antibody and titered on hepatoma cells (*n* = biological replicates). **d** Relative infectivity of HEV particles recovered from the lysate or released in the supernatant of hepatoma cells transfected with HEV RNA from strain Kernow-C1 P6, 7 d post-transfection. Harvested HEV particles were treated with 1:100 anti-ORF2 antibody, IgG-control antibody and/or 0.1% sodium deoxycholate (DOC) for 30 min at RT. HEV infectivity was determined by titration on hepatoma cells (*n* = biological replicates). **e** Intra- and extracellular HEV particles from HEV P6 RNA-transfected hepatoma cells were mixed with either apical or basal supernatant from pol-HLCs and/or 1:100 anti-ORF2 antibody prior to titration on hepatoma cells (*n* = biological replicates). **f** Western blot analysis of cell lysates from shCYP8B1-inducible, H9-derived pol-HLCs (H9/shCYB8B1). Cells were treated or untreated for 48 h with 3 μg/ml doxycycline (DOX) to induce shRNA expression. Shown are representative images of *n* = 2. **g** Apical total bile acid release from H9/shCYP8B1-derived pol-HLCs treated with 3 μg/ml DOX as indicated (*n* = biological replicates). **h** Extracellular HEV particles from HEV P6 RNA-transfected S10-3 cells were mixed with apical supernatant from H9/shCYP8B1-derived pol-HLCs treated with or without 3 μg/ml DOX (*n* = biological replicates). **i** HEV secretion model from non-polarized and polarized HLCs. **j** Pol-HLCs were infected with HAV strain HM175/18 f. 48 h post infection, newly released particles were harvested and titered on hepatoma cells (*n* = biological replicates). Statistical analysis was performed using a two-tailed unpaired *t* test with Bonferroni adjustment for multiple comparisons.

We previously showed that nonpol-HLCs are permissive for HEV infection and replication, but few infectious progeny particles were released^21^. To test if pol-HLCs support HEV infection, we infected the cells from their basolateral side, harvested newly secreted virus from either the apical or basolateral compartment at 7 days post infection, and measured infectivity on human hepatoma S10-3 cells (Fig. 4a). To confirm replication-dependent secretion of new particles, we treated pol-HLCs with either sofosbuvir (SOF), an HEV RNA replication inhibitor^30^, or a dimethyl sulfoxide (DMSO) vehicle control. Newly produced infectious particles were released into both compartments, but the majority (fivefold more) was released on the apical side (Fig. 4a). To assess whether this was also the case for physical virus particles, we quantified HEV RNA copies. As shown in Fig. 4b, pol-HLCs released threefold more physical particles from the apical membrane, which did not fully account for the fivefold increased infectivity. This suggested that apically released particles were more infectious than the basolaterally released particles. We also observed a higher amount of capsid protein ORF2 released from the apical membrane. The ELISA used in this study does not distinguish between the glycosylated and non-glycosylated ORF2 forms that have been recently found to be associated with infectious particles^31,32^. The possible directional release of these two distinct ORF2 forms can be now analyzed with the pol-HLC system.

The quasi-envelope on HEV particles has been previously shown to reduce their infectivity^33^. In cell culture, non-polarized hepatoma cells release only quasi-enveloped particles (extracellular HEV, Fig. 4i). As they are not polarized, these cells must be lysed to recover non-enveloped particles (intracellular HEV). In agreement with previous studies^28,33–36^ we found that the quasi-envelope rendered extracellular HEV less infectious (Supplementary Fig. 5), but protected the virus from neutralization by anti-HEV capsid ORF2 protein antibodies (Fig. 4d). Unlike apically released particles, basolaterally released particles were fully protected from neutralization with anti-ORF2 (Fig. 4c) and could not be immunoprecipitated with an anti-ORF2 antibody (Supplementary Fig. 6). This confirmed the presence of the quasi-envelope, which rendered basolateral HEV particles less infectious. Our data are therefore in agreement with observations made in vivo and the current model for polarized HEV particle production of naked (apical) highly infectious virions and quasi-enveloped (basolateral) less infectious, neutralization-resistant particles (reviewed in refs. ^37,38^).

As treating extracellular HEV with detergent or bile salts removes the quasi-envelope^28,34^ (Fig. 4d), we tested whether apically secreted bile salts from pol-HLCs (Fig. 3e) could transform basolaterally released HEV into non-enveloped and highly infectious particles. Extra- and intracellular HEV particles harvested from HEV replicating S10-3 cells were treated with either apical or basolateral supernatant from pol-HLCs (Fig. 4e). Apical but not basolateral supernatant enhanced the infectivity of extracellular but not intracellular HEV particles. Once treated with apical supernatant, extracellular particles could be readily neutralized with anti-ORF2 (Fig. 4e); albeit with a lower efficiency, possibly owing to insufficient bile acid concentration.

To further examine the role of bile acids, we transduced H9 cells with a lentivirus delivering a doxycycline (DOX) inducible short hairpin RNA (shRNA) targeting *CYP8B1*, a key enzyme responsible for converting cholesterol to bile acid (H9/shCYP8B1). After selecting transfected cells, we differentiated H9/shCYP8B1 cells to pol-HLCs. Forty-eight hours before terminal HLC differentiation, we treated H9/shCYP8B1 cells with 3 μg/ml DOX to induce shRNA expression and knockdown of CYP8B1. As a result, CYP8B1 expression (Fig. 4f) and apical bile acid release (Fig. 4g) were reduced by ~30%. We then mixed apical supernatant from these cells with extracellular HEV particles (Fig. 4h & Supplementary Fig. 7). Unlike supernatant from wild-type cells, this apical supernatant failed to enhance infectivity of extracellular HEV particles, suggesting that the co-secreted bile acids from pol-HLCs were responsible for removing the envelope (Fig. 4i).

We also used pol-HLCs to study the directional secretion of HAV, which similar to HEV, is enterically transmitted and acquires a quasi-envelope during budding^39^. HAV efficiently infected pol-HLCs (Supplementary Fig. 8). However, the HAV strain used in our study was cytolytic for pol-HLCs disrupting the cellular monolayer by 48 h post infection. This is likely the reason why similar levels of HAV were found in both transwell compartments after 48 h of infection (Fig. 4j). In order to study directional HAV release, it will be interesting to examine natural HAV isolates, which do not appear to lyse infected cells^40^.

The pol-HLC system can thus be infected with two enterically transmitted hepatitis viruses, HAV and HEV, providing a platform to analyze and compare their mechanisms of assembly and secretion from polarized hepatocytes.

### Drug uptake, metabolism, and secretion

Many drugs are metabolized and eliminated by hepatocytes. Hepatocytes have phase I and III enzymes that metabolize compounds, which are then secreted by phase III drug transporters into either the blood (from the basolateral membrane) or into bile (from the apical membrane). Hepatobiliary elimination can be a major clearance pathway that dictates drug pharmacokinetics. In vitro, current available cellular models to study drug disposition are limited. PHH in sandwich cultures are the gold standard for these studies, but they secrete apical cargo into bile pockets, which must be accessed to measure biliary efflux and evaluate biliary transporter interactions. This is technically challenging and moreover, opening the bile canaliculi destroys the cells, which precludes simple non-disruptive measurements of apical drug release over time.

**Fig. 5:**
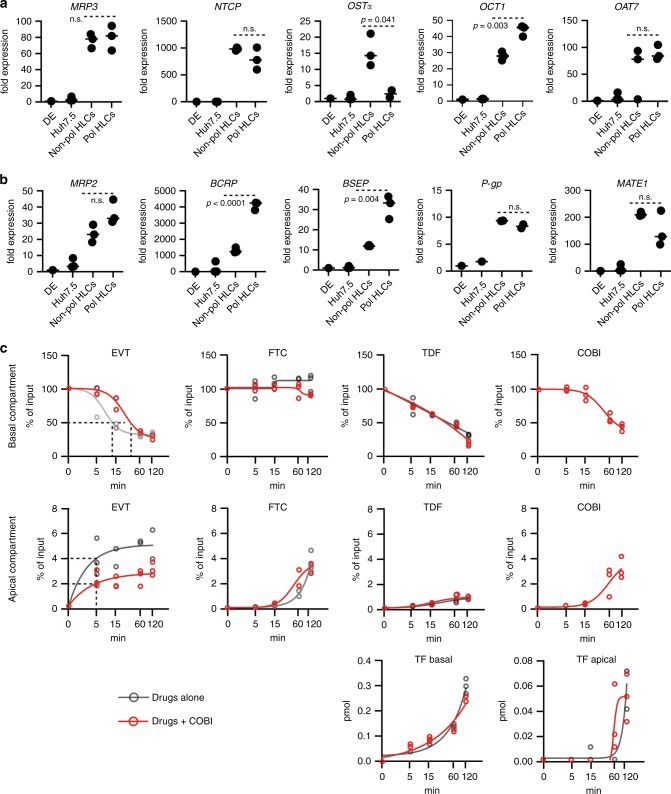
Polarized HLCs for drug–drug interaction, absorption, and secretion studies. Relative mRNA levels quantified by RT-qPCR of (**a**) basolateral and (**b**) apical transporters in DE, Huh7.5, nonpol-, and pol-HLCs. Statistical analysis was performed using a two-tailed unpaired *t* test (*n* = biological replicates). **c** Modeling stribild disposition in pol-HLCs. Pol-HLCs were incubated with 10 μm elvitegravir (EVT), 13 μm emtricitabine (FTC), 20 μm tenofovir disoproxil fumarate (TDF) ± 10 μm cobicistat (COBI) from the basolateral compartment. At the indicated time points, compounds and the TDF metabolite tenofovir (TF) were quantified by LC-MS in apical and basolateral compartments (*n* = biological replicates).

As the pol-HLC system has constrained apical and basolateral compartments and secretes accessible cargo directionally, we tested the system for studying antiviral drug absorption, vectorial efflux, and drug–drug interactions. Numerous studies proposed the use of non-polarized HLCs to screen drugs and study drug-induced toxicity^6,10,41–43^. We first compared pol-HLCs with nonpol-HLCs for their ability to express enzymes and transporters that mediate drug disposition. RNA-Seq analysis revealed that phase I and II drug-metabolizing enzymes were differentially expressed by pol- and nonpol-HLCs (Supplementary Fig. 9). As shown in Fig. 5a and b and in agreement with the RNA-Seq data (Fig. 3), pol-HLCs expressed higher levels of apical, but not basolateral phase III drug transporters. This suggested that pol-HLCs were enhanced in their ability to mediate hepatobiliary drug secretion.

We then tested pol-HLC for modeling an antiviral combination drug regimen that has well characterized pharmacokinetics (Fig. 5c). For this we chose STRIBILD, a fixed dose anti-HIV combination therapy. This regimen includes emtricitabine (FTC), tenofovir disoproxil fumarate (TDF), elvitegravir (EVT), and cobicistat (COBI). To model STRIBILD disposition, we incubated pol-HLCs from their basolateral membrane with FTC, TDF, and EVT combined at a ratio mimicking the approved antiviral regimen with or without COBI. Unlike the other three compounds, which are direct virus replication inhibitors, COBI blocks the drug-metabolizing CYP3A4 enzymes, as well as drug transporters. We then measured the absorption and secretion from either basolateral or apical membrane over a 2 h period using LC-MS. As shown in Fig. 5c, the cells absorbed each drug at a different rate, as evidenced by the steady decrease of free compound in the bottom transwell compartment. EVT and TDF were rapidly absorbed, whereas FTC, which is known to have a long plasma half-life^44^, was slowly absorbed. Concomitant with the decrease in the bottom transwell compartment, the amount increased in the top compartment, suggestive of partial biliary release from the apical membrane. We also detected a slow release of tenofovir (TF) in both compartments, demonstrating that pol-HLCs metabolized TDF converting it to TF. Co-administering COBI only affected EVT absorption, which unlike FTC and TDF, is metabolized by the CYP3A4 enzyme family. COBI decreased the rate of EVT absorption by approximately threefold and reduced apical EVT release by ~50%. This is in agreement with EVT’s increased plasma half-life (from 3–9 h) when boosted by COBI, allowing it to be dosed once daily^45,46^. These observations show that COBI helps sustain basolateral levels of EVT in blood by reducing its uptake and metabolism in hepatocytes. In contrast, COBI did not affect FTC or TDF levels, which suggested that the observed decrease of EVT is not owing to diffusion between the two transwell compartments but rather is limited by the action of enzymes and transporters. Therefore, the pol-HLC system recapitulates observations made in humans and demonstrates the potential application to pre-clinical drug development^46^.

## Discussion

Cell polarity is based on the asymmetric organization of cellular components, and is a pre-requisite for fundamental biological processes. It enables a polarized cell to ensure directional cargo transport and release while maintaining a barrier within the epithelium. Although most epithelial cells typically establish a columnar apical-basal polarity, hepatocytes distinguish themselves by their multipolar organization, which allows them to exert their particular activities in the 3D environment of the liver. Current in vitro models for hepatocyte polarity studies are suboptimal. Only few hepatoma cell clones can be columnar polarized, but their transformed nature makes them undesirable for many applications. Transferring already differentiated, non-proliferative PHHs onto the transwell membrane does not give rise to PHH with columnar polarization but rather induces restoration of their previously formed multipolar structure, which restricts apical and basal cargo sampling (data not shown). Similarly, plating human fetal hepatoblasts (usually from week 15 to 21 of gestation) from different donors onto the transwell membrane also fails to form a tight monolayer and columnar polarized hepatocytes (data not shown). Here, we report a stem cell-based differentiation protocol to generate polarized HLCs on transwell filters. The use of transwell filters yielded a columnar polarization that was likely achieved and supported by the one-sided exposure to ECM and appropriate nutrient gradients.

We first examined the structural polarization of pol-HLCs by analyzing the localization of hepatocyte membrane proteins. Immunofluorescence staining showed that pol-HLCs have clearly defined basolateral and apical membranes separated by tight junctions. We also showed that pol-HLCs possess functional polarity. Hepatic cargo destined to reach the bloodstream such as albumin, urea, and lipoproteins were secreted basolaterally. In contrast, bile acids, destined for secretion into the biliary system, were secreted apically from pol-HLCs. We further found that pol-HLCs can recapitulate the directionality of HEV infection in vivo: HEV progeny particles are secreted basolaterally as quasi-enveloped particles and apically as naked virions. Knocking down *CYP8B1*, a key enzyme in bile acid metabolism, revealed that co-secreted bile acids strip the envelope from apically released virions rendering them highly infectious. Finally, by showing that pol-HLCs faithfully replicated hepatocyte uptake and biliary/blood excretion of the once-daily anti-HIV regimen STRIBILD, we provided proof-of-concept that polarized HLCs can be used for pharmacokinetic and drug–drug interaction studies.

We are aware that despite recapitulating some hepatocyte functions better than nonpol-HLCs, pol-HLCs still retain an immature phenotype as evidenced by the transcriptome comparison with PHHs (Supplementary Fig. 2). Efforts are ongoing by multiple labs to optimize protocols and identify compounds and conditions that can enhance maturation of HLCs. Success and combination with the protocol described here may eventually yield highly functional polarized HLCs that better recapitulate fully mature hepatocyte functions. However, the system described here has several immediate advantages. Unlike PHHs, pol-HLCs are derived from a renewable, reproducible, and cost-effective source. Furthermore, the resulting apical-basal polarity and release of cargo in separate compartments allows easy sampling and analysis over time. This, together with the ability to genetically manipulate cellular proteins of interest such as membrane transporters, will help advance our general understanding of the polarized hepatic trafficking machinery and how components are selectively targeted to the apical versus the basolateral compartment.

Members of the CYP450 family and other phase I, II, and III drug-metabolizing enzymes have poor expression and induction levels in hepatoma cells, which is why they are unsuitable for drug metabolism and disposition studies. As mentioned before, HLCs have been proposed as an attractive alternative to overcome these limitations. In addition, as HLCs can be generated from patient-specific iPSCs^47^, studying the impact of genetic polymorphism and inter-individual variations on drug exposure and toxicity can facilitate the development of personalized therapies. The ability of pol-HLCs to excrete drugs from either the basolateral or apical membrane will improve these types of studies.

In conclusion, this novel stem cell differentiation protocol provides a powerful cell culture system to study proper hepatocyte function, which will lead not only to a better understanding of normal liver physiology, but also holds promise for informing therapeutic options and drug development.

## Methods

### Reagents and antibodies

The following antibodies were used for immunofluorescence staining or western blot analyses: anti-FoxA2 (used at 1:400, Cell Signaling), anti-HNF4α (used at 1:500, Cell Signaling), anti-AFP (used at 1:1000, Sigma-Aldrich), anti-ALB (used at 1:1000, Cedarlane, Burlington, Canada), anti-ZO-1 (used at 1:1000, Thermo Fisher), anti-E cadherin (used at 1:500, Cell Signaling), anti-SR-BI (used at 1:100, Novus Biologicals), anti-BCRP (used at 1:100, Millipore), anti-MRP2 EAG5^48^ (used at 1:200, a kind gift from Anne Nies, IKP Stuttgart^48^), anti-Apo-CIII (used at 1:500, Abcam), anti-CYP8B1 (used at 1:100, Abcam), anti-ORF2 (used at 1:400, a kind gift from Suzanne U. Emerson, NIH) and anti-HAV capsid (used at 1:1000, a kind gift from Stanley M. Lemon, UNC School of Medicine). Alexa Fluor 488 and 549 anti-mouse (used at 1:1000) and Alexa Fluor 488 and 549 anti-rabbit (used at 1:1000) antibodies were purchased from ThermoFisher. Alexa 594-conjugated transferrin was purchased from ThermoFisher. Tenofovir, Tenofovir disoproxil fumarate, and Emtricitabine were obtained through the AIDS Reagent Program, Division of AIDS, NIAID, NIH. Elvitegravir and Cobicistat were purchased from SelleckChem. Sofosbuvir was purchased from Acme Bioscience. BX795, oleic acid and lomitapide were purchased from Sigma-Aldrich.

### Polarized HLC stem cell differentiation

In all, 2×10^5^ cells/cm^2^ of ESC or iPSC were differentiated to DE by harvesting them with gentle cell dissociation reagent (Stemcell Technologies) and plating onto Matrigel (Corning, Catalog number 354230)-coated culture dishes (Corning) in mTeSR1 medium (Stemcell Technologies). The next day, DE differentiation was initiated by using the STEMdiff Definitive Endoderm Kit (Stemcell Technologies). To induce hepatic differentiation, DE cells were harvested using Accutase (Innovative Cell Technologies), re-seeded in Matrigel-coated transwells (Transwell, Corning, Catalog number 3460) as described in Results and cultured in the presence of medium A (basolateral medium (BM): CTS KnockOut DMEM/F12, 10% KnockOut Serum Replacement, 0.5% GlutaMAX supplement, and 0.5% non-essential amino acids all from ThermoFisher Scientific, supplemented with 100 ng/ml HGF), 1% DMSO) for 8 days followed by incubation in medium B (BM, 100 ng/ml HGF, 1% DMSO, 40 ng/ml dexamethasone) for three 3 days. Cells were further matured in HCM (Lonza, omitting the EGF) supplemented with 20 ng/ml oncostatin-M for 5–7 days. For nonpol-HLCs, DE cells were seeded on Matrigel-coated culture plates and step-wise matured in the media described above. HGF was purchased from Peprotech, dexamethasone from Sigma, and oncostatin-M from R & D Systems.

### Isolation and culture of PHHs

PHHs were isolated from patient liver tissue after partial hepatectomy. The protocol was authorized following written informed consent of the patients and approved by the ethics commission of Hannover Medical School (EthikKommission der MHH, Nr. 252-2008). Isolated PHHs were seeded on collagen-plated plates and used for studies 48 h post plating.

### RT-PCR and real-time quantitative RT-PCR

Total RNA was isolated from cell lysates using the RNeasy Mini Kit (Qiagen) followed by reverse transcription using Superscript III Reverse Transcriptase (Thermo Fisher Scientific). Gene expression was quantified using the LightCycler SYBR Green I Master mix (Roche Life Science, Indianapolis, IN) on a LightCycler 480 Instrument I (Roche Life Science) with primers as listed in Supplemental Table 1. Relative expression data were calculated into fold changes based on cycle thresholds.

### Fluorescent dextran assay

Polarized HLCs were incubated with 4 kDa fluorescein isothiocyanate–dextran (Sigma-Aldrich) or 70 kDa rhodamine B isothiocyanate–dextran (Sigma-Aldrich) diluted in either apical or basolateral HCM medium to a final concentration of 1 mg/ml overnight (O/N) at 37 °C. To dissociate cell–cell junctions, 2.5 mm EDTA (Gibco) was added. Fluorescence was measured using a FLUOstar Omega plate reader (BMG Labtech) (FITC-dextran: Exc: 485 nm and Em: 544 nm and rhodamine B-dextran: Exc: 520 nm and Em: 590 nm).

### Transmission electron microscopy

Cells were fixed with 2% paraformaldehyde (PFA) and 2.5% glutaraldehyde in 0.075 m sodium cacodylate buffer pH 7.4. Subsequently, cells were washed in the buffer, post-fixed with 1% osmium tetra-oxide for 1 hr, underwent en bloc staining with 1% uranyl acetate for 30 min, dehydrated by a graded series of ethanol, infiltrated with a resin (Eponate12, Electron Microscope Sciences) and embedded with the resin. After polymerization at 60 °C for 48 h, ultra-thin sections were cut, underwent post-staining with 2% uranyl acetate and 1% lead citrate and were examined under a JEOL JEM 1400Plus transmission electron microscope equipped with SerialEM^49^ in montage mode and the digital imaging system (Gatan Digital Micrograph 1000) (a gift from the Helmsley Charitable Trust). EM data were processed by IMOD^42^.

### Immunofluorescence staining and analysis

Cells were fixed in 4% PFA in phosphate-buffered saline (PBS) at room temperature (RT) for 30 min and blocked with 3% bovine serum albumin in PBS (3% B-PBS). Cells were incubated with primary antibodies in 3% B-PBS at 4 °C overnight. Secondary antibodies conjugated to Alexa Fluor 594 (Thermofisher) or Alexa Fluor 488 (Thermofisher) in 3% B-PBS were added and incubated at RT for 1 hr, followed by several washes with PBS. The staining with transferrin-594 conjugate (Thermofisher) was performed following the manufacturer’s protocol. For HLC cultures grown on transwell filters, the filter was removed from the hanging insert and submerged, cell side up, in PBS using a slice anchor (Warner Instruments). Cultures were imaged on an Upright BX61WI microscope (Olympus) with a UMPlan FL 60 × 1.0 NA water dipping objective (Olympus) or UMPlan FL ×10, 0.3 NA water dipping objective and an Orca Flash 4.0 digital CMOS camera (Hamamatsu) using MetaMorph image acquisition software (Molecular Devices). Deconvolution was performed using the standard adaptive point spread settings in Autoquant (Media Cybernetics). Image analysis was conducted using Fiji.

### RNA-seq analysis

Approximately 100 ng of total RNA isolated from biological duplicate samples using the RNeasy mini Kit (Qiagen) was used as input. Sequencing libraries were constructed using the TruSeq Stranded mRNA LT Sample Prep Kit (Illumina) and sequenced on an Illumina HiSeq with a read length of 51 nt (single end, reverse stranded). Output sequencing read quality was analyzed using the Seqtk (version 1.2) and fastx_toolkit (version 0.0.14) software tools and used without further processing. The reads were mapped to the Ensembl human genome assembly GRCh37 (also known as hg19) using Tophat2 (version 2.0.12; Bowtie version 2.2.7) with first-strand library type, no novel junctions, and otherwise default options. Mapped reads were counted using featureCounts (from subread version 1.4.6) with default options. Statistical analysis was performed using a count-based workflow^50^ with the edgeR Bioconductor package (version 3.12.1). In brief, gene counts were normalized to counts per million reads and genes with <10–15 mapped reads were filtered out. Transcriptome composition bias between samples was eliminated using edgeR’s default trimmed mean of M-values normalization. Differential expression analyses were performed using edgeR’s quantile-adjusted conditional maximum likelihood method.

### Western blotting

Cell lysates were separated by 10% sodium dodecyl sulfate-polyacrylamide gel electrophoresis, followed by transfer onto polyvinylidene fluoride membrane (EMD Millipore, Billerica, MA), as previously described^30^. Western blotting analysis was performed using specific antibodies as described above.

### Total bile acids assay

Bile acids were measured using the total bile acid assay kit from Cell Biolabs, Inc. following the manufacturer’s protocol.

### Bile acid quantitation by LC-MS

An Agilent 1290 UHPLC coupled to a Sciex 4000 QTRAP mass spectrometer was used. The MS instrument was operated in the multiple-reaction monitoring (MRM) mode with negative-ion (−) detection. A Waters BEH C18 column (2.1 mm I.D. × 15 cm, 1.7 μm) was used for LC separation with a mobile phase composed of (A) 0.01% formic acid in water and (B) 0.01% formic acid in acetonitrile for binary-solvent gradient elution. For the reference substances, a standard mix containing all targeted bile acids was dissolved in 50% methanol at 10 nmol/ml. This solution (S1) was further diluted step by step at a same dilution ratio of 1–4 (v/v) with the same solvent to have standard solutions of S2 to S10. 50 μl of S1 to S10 each was mixed with 50 μl of an internal standard (IS) solution containing 14D-labeled bile acid analogs. In all, 20 μl of each solution was injected to run UPLC-(−)ESI-MRM/MS. Linear calibration curves were constructed using analyte-to-internal standard peak area ratios (As/Ai) versus molar concentrations (nmol/mL) of each bile acid. For the samples, 50 μl of each vortex-mixed cell medium was mixed with 25 μl of the IS solution and 250 μl of acetonitrile in an Eppendorf tube. After sonication in an ice-water bath for 2 min, the mixture was centrifuged at 15,000 rpm at 10 °C for 15 min. The clear supernatant was transferred into a “V”-shape LC injection microvial and dried in a speed-vacuum concentrator. The residue was reconstituted in 100 μl of 50% methanol. 20 μl was injected for quantitation of bile acids by UPLC-(−)ESI-MRM/MS.

### Lipoprotein secretion assay

Pulse-chase labeled lipoprotein secretion assays and density gradient separation were performed as previously described^19^. In brief, cells were metabolically labeled for 4 h with [^35^S] methionine/cysteine. Supernatants from labeled cells were subjected to density gradient centrifugation followed by ApoB100 or ApoE immunoprecipitation of each fraction, separation by SDS-PAGE, and detection by [^35^S] fluorography.

### ELISAs

HEV ORF2 (Wantai), ALB, ApoB and ApoB ELISAs (Abcam) were performed according to manufacturer’s protocols.

### Generation of Apo-CIII-KO cells

Guide RNA sequence (5′-GCACGCCACCAAGACCGCCA-3′) targeting exon 2 of ApoC-III locus was cloned into the pX458 vector, a gift from Feng Zhang (Addgene plasmid #48138), in vitro transcribed using the MEGAshortscript Kit, and purified using the MEGAclear Kit (Thermo Fisher Scientific). The guide RNA was transfected into HUES8-iCas9wt cells, which upon treatment with 3 μg/ml Dox inducibly express CRISPR-Cas9^14^. Dox was added to transfected cultures for 48 h and cells were seeded in clonal limiting dilution and grown on irradiated mouse embryonic fibroblasts. ApoC-III-KO cells were screened by and validated by genome sequencing and Western Blot analysis for absence of ApoC-III expression.

### HEV and HAV infections

Cell culture grown HEV strain Kernow-C1 P6 (GenBank accession number JQ67901) and HAV strain HM175/18 f (GenBank accession number M59808) were generated as previously described^30,51^. Polarized HLCs were infected with cell culture grown HEV or HAV at day 21 of the differentiation protocol by inoculation from the basolateral membrane. Virus inoculum was removed the next day, followed by washes with HCM medium. Cells were then maintained in HCM medium, with medium renewals every second day. Newly secreted virus was harvested from either the apical or basolateral compartment (7 days post infection for HEV and 2 days post infection for HAV for titration) on S10-3 hepatoma cells (a generous gift from Suzanne U. Emerson, NIH).

### Drug disposition analysis by LC-MS/MS

Samples were submitted in a 10 μl volume of which 3 μl (30% of total) was analyzed by liquid chromatography tandem mass spectrometry (LC-MS/MS, SRM). Compounds were separated by reversed phase chromatography (C_8_, 150 mm length × 2.1 mm ID, Acclaim Thermo) using a gradient delivered at 200 μL/min, increasing from 5% B/95% to 50% B/50% A in 5.5 min. (Solvent A: 0.1% Formic Acid, Solvent B: Acetonitrile/0.1% formic acid). The most intense fragment ion was used as transition for each compound when quantitated: Cobicistat: 776.4^+^→606.2^+^, Elvitegravir: 448.0^+^→344.0^+^, Emtricitabine: 248.0^+^→130.1^+^, and 270.0^+^ (sodium ion adduct) →152.0^+^, Tenofovir: 520.1^+^→270.0^+^, and Fluoxetine as internal standard: 310.0^+^→148.0^+^. Each molecule was quantitated using an external calibration (12 fm, 24 fm, 48 fm, 98 fm, 195 fm, 0.78 pm, 1.56 pm, 3.13 pm, 6.25 pm, 12.5 pm, 25.0 pm, 50.0 pm, and 100 pm). Dwell time of 50 msec together with an isolation of 0.7 Th was used for all compounds.

### Statistics

 Graphs and statistical analyses were performed using GraphPad PRISM 5 (GraphPad Software Inc.). In all figures where *p*-values were calculated, the corresponding statistical test is listed in the figure legend. 

### Reporting summary

Further information on research design is available in the Nature Research Reporting Summary linked to this article.

## Supplementary information

Supplementary information

Reporting summary

## Data Availability

Full scans of the blots are available in the Source Data file. The RNA-seq data sets have been deposited in NCBI’s Gene Expression Omnibus and are accessible through GEO Series accession number GSE123462.

## Source data

Source data

## Code availability

Code used as part of the work presented in this manuscript is available from the authors upon reasonable request.
