# Supplementary material for: Stem cell-derived polarized hepatocytes
Source: Nat Commun. 2020 Apr 3;11:1677. doi: 10.1038/s41467-020-15337-2 (PMC7125181; doi:10.1038/s41467-020-15337-2)
Supplement: Supplementary file 1 — Supplementary information [file 41467_2020_15337_MOESM1_ESM.pdf]

Supplementary Information

Stem cell-derived polarized hepatocytes

Dao Thi, VL et al.

Supplementary Figure 1

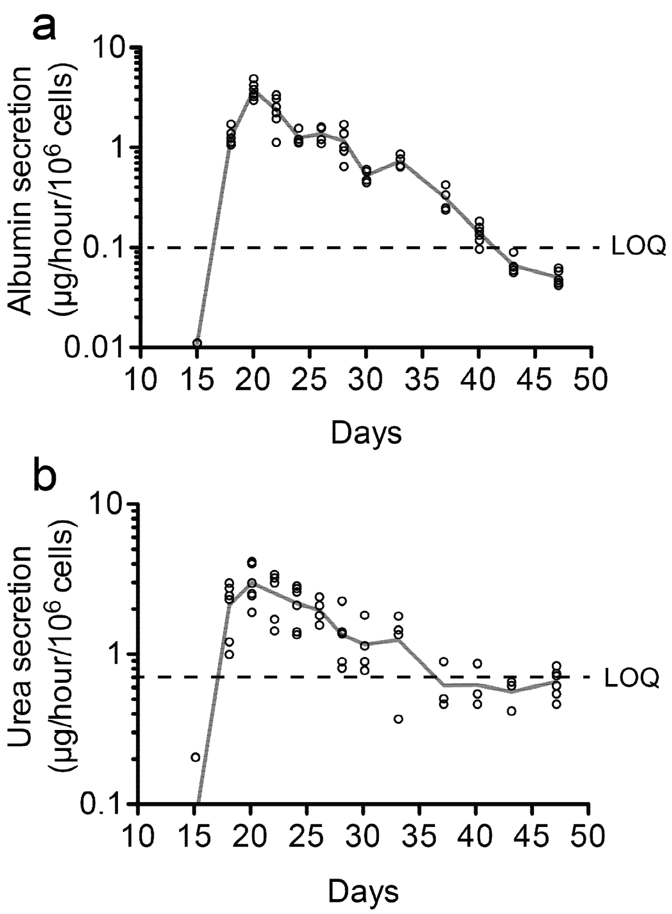

Supplementary Figure 1: Pol-HLCs remain stable for up to three weeks in culture. (a) Albumin and **(b)** urea were measured in basal supernatants from pol-HLCs over indicated period of time.

Supplementary Figure 2

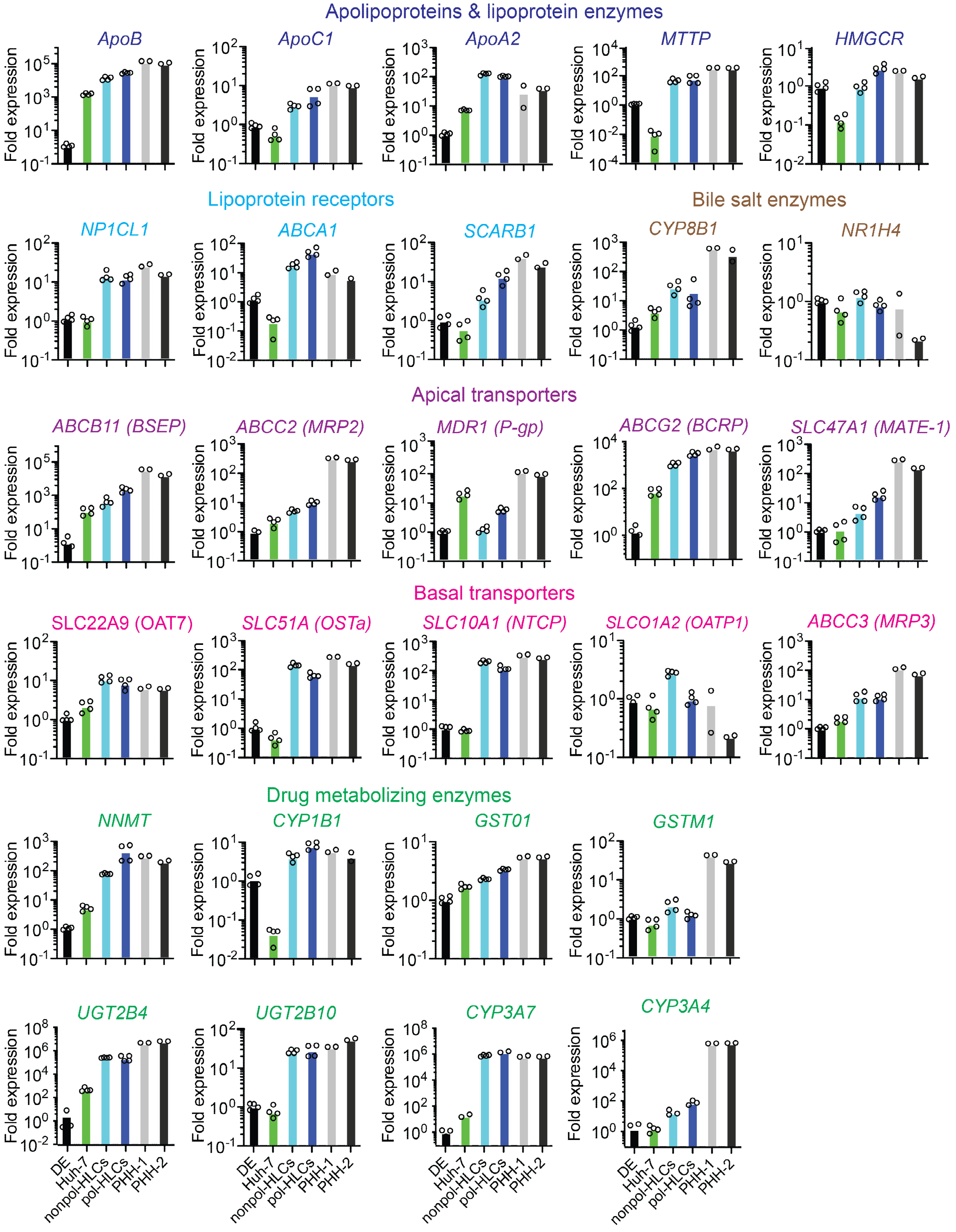

Supplementary Figure 2. Comparison of gene expression in different hepatocyte types.
Relative mRNA levels quantified by RT-qPCR of indicated genes in lysates of definitive endoderm cells (DE), Huh-7, nonpol-HLCs, pol-HLCs and PHH.

Supplementary Figure 3

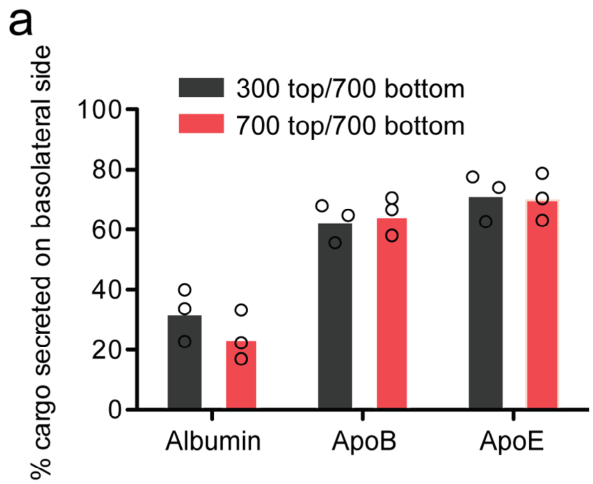

Supplementary Figure 3. Vectorial cargo release from polarized HLCs. Relative amounts of albumin, ApoB, and ApoE released by pol-HLCs in bottom compartment were quantified by ELISA. Cells were either incubated with 300 µl or 700 µl hepatocyte culture medium (HCM) from the top, as indicated, and 700 µl HCM from the bottom.

Supplementary Figure 4

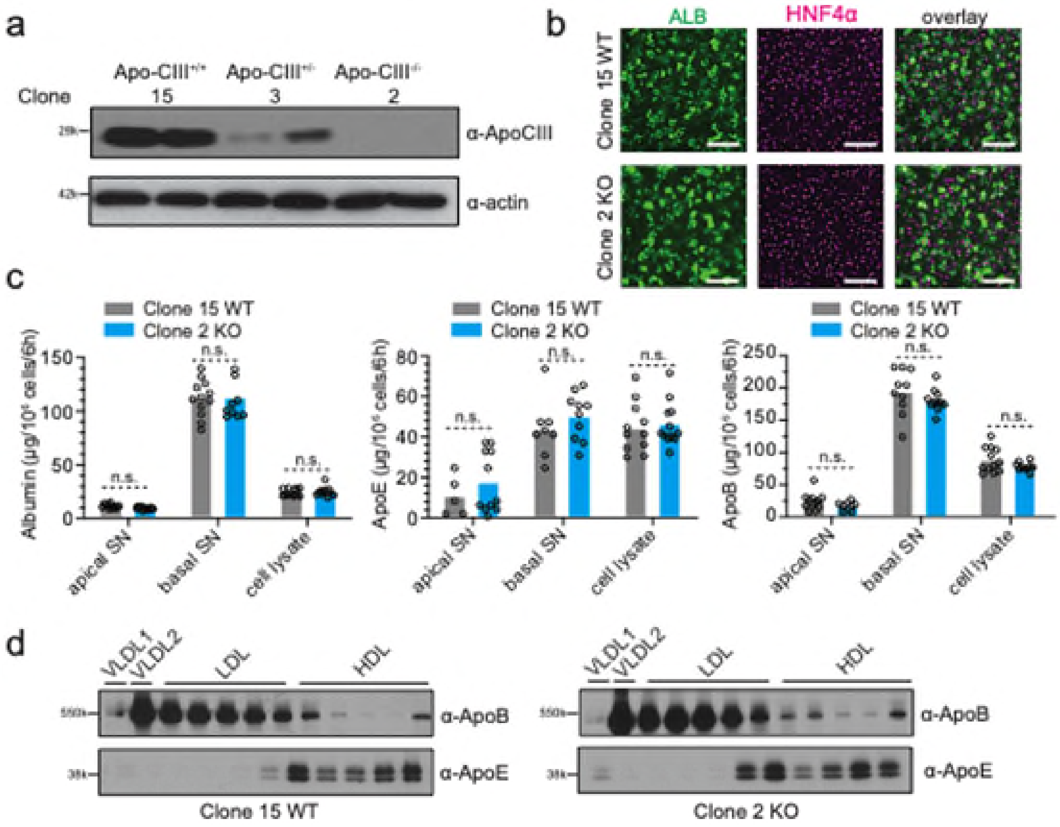

Supplemental Figure 4. Apo-CIII knockout does not alter very low-density lipoprotein assembly and release. (a) Western blot analysis of HUES8-iCas9wt-derived clones Apo-CIII$^{+/+}$ clone 15, Apo-CIII$^{+/-}$ clone 3 and Apo-CIII$^{-/-}$ clone 2 cell lysates differentiated to pol-HLCs in duplicate were used to probe Apo-CIII and β-actin. **(b)** Apo-CIII$^{+/+}$ clone 15- and Apo-CIII$^{-/-}$ clone 2-derived pol-HLCs were stained for hepatocyte marker ALB (green) and HNF-α (magenta). **(c)** ELISA quantification of albumin, ApoB100 and ApoE released during 6hrs or recovered from lysates of pol-HLCs derived from indicated cell clone. Statistical analysis was performed using a two-tailed unpaired t-test. **(d)** Density distribution of ApoB100 and ApoE secreted from pol-HLCs derived from indicated cell clone. Cells were incubated with oleic acid for 3 hrs followed by metabolic labeling for 3 hrs with [^{35}S]methionine/cysteine. Images are representative of three independent differentiations.

Supplementary Figure 5

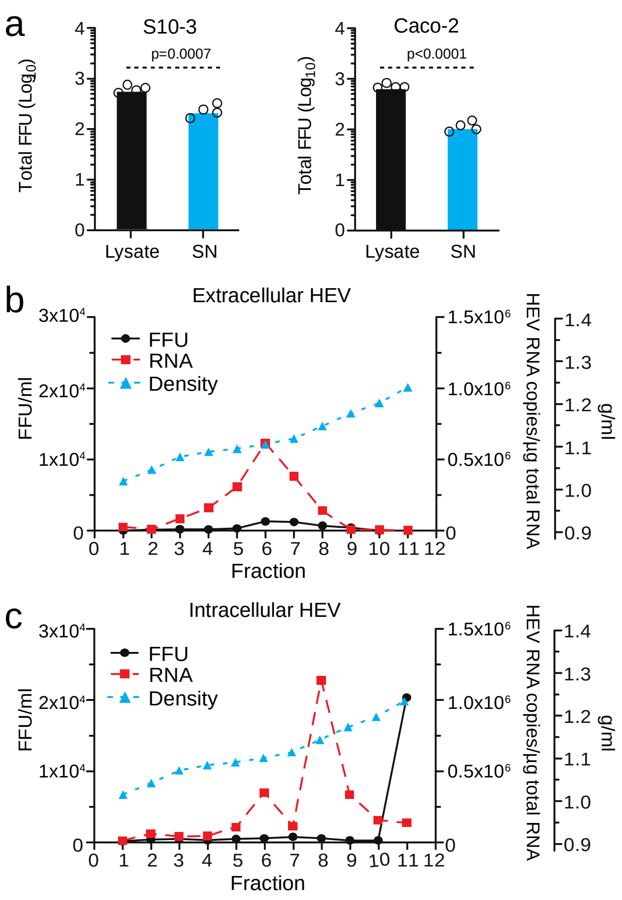

Supplementary Figure 5. Intra- and extracellular cell culture grown HEV particles have differential properties. (a) Infectious HEV particles recovered from supernatant or lysates of $1x10^5$ hepatoma S10-3 cells transfected with 1 µg full-length HEV P6 Kernow-C1 gt3 RNA titrated on either S10-3 (left panel) or intestinal Caco-2 cells (right panel). Statistical analysis was performed using a two-tailed unpaired t-test. Quantification of **(b)** extra- and **(c)** intracellular HEV particles separated by density centrifugation for infectivity and genome equivalents.

Supplementary Figure 6

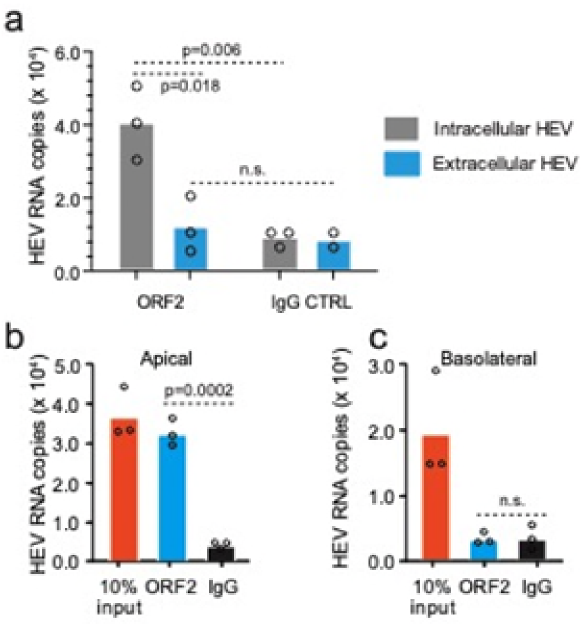

Supplementary Figure 6: Immunoprecipitation of naked and quasi-enveloped HEV particles. (a) Infectious HEV particles recovered from supernatant or lysates of $1x10^6$ hepatoma S10-3 cells transfected with HEV P6 Kernow-C1 gt3 RNA or **(b)** HEV particles released from HEV infected pol-HLCs were immunoprecipitated with an anti-ORF2 or IgG control antibody and quantified by qRT-PCR. Statistical analysis was performed using a two-tailed unpaired t-test.

Supplementary Figure 7

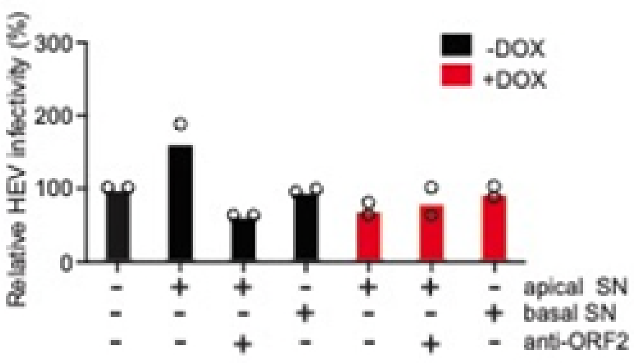

Supplementary Figure 7. Apical supernatant from pol-HLCs with downregulated CYP8B1 fails to enhance extracellular HEV particle infectivity. Extracellular HEV particles from HEV P6 RNA-transfected S10-3 cells were mixed with either apical or basal supernatant from H9/shCYP8B1-derived pol-HLCs treated with or without 3 µg/ml DOX.

Supplementary Figure 8

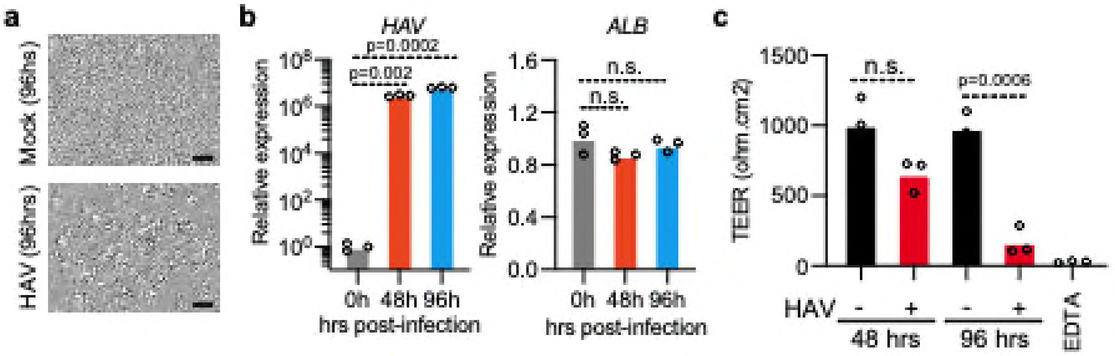

Supplementary Figure 8. Infection with Hepatitis A virus strain HM175/18f disrupts the integrity of pol-HLCs. (a) Brightfield image of HAV-infected or mock treated pol-HLCs 96 hrs post-infection. Shown are representative images of n=2. Scale bars = 50 µm **(b)** HAV genome copies and relative ALB mRNA levels were quantified in lysates of infected cells at indicated time points post-infection by qRT-PCR. **(c)** Transepithelial electrical resistance (TEER) of HAV-infected pol-HLCs was measured at indicated time points. EDTA-treated pol-HLCs were used as positive control. Statistical analysis was performed using a two-tailed unpaired t-test.

Supplementary Figure 9

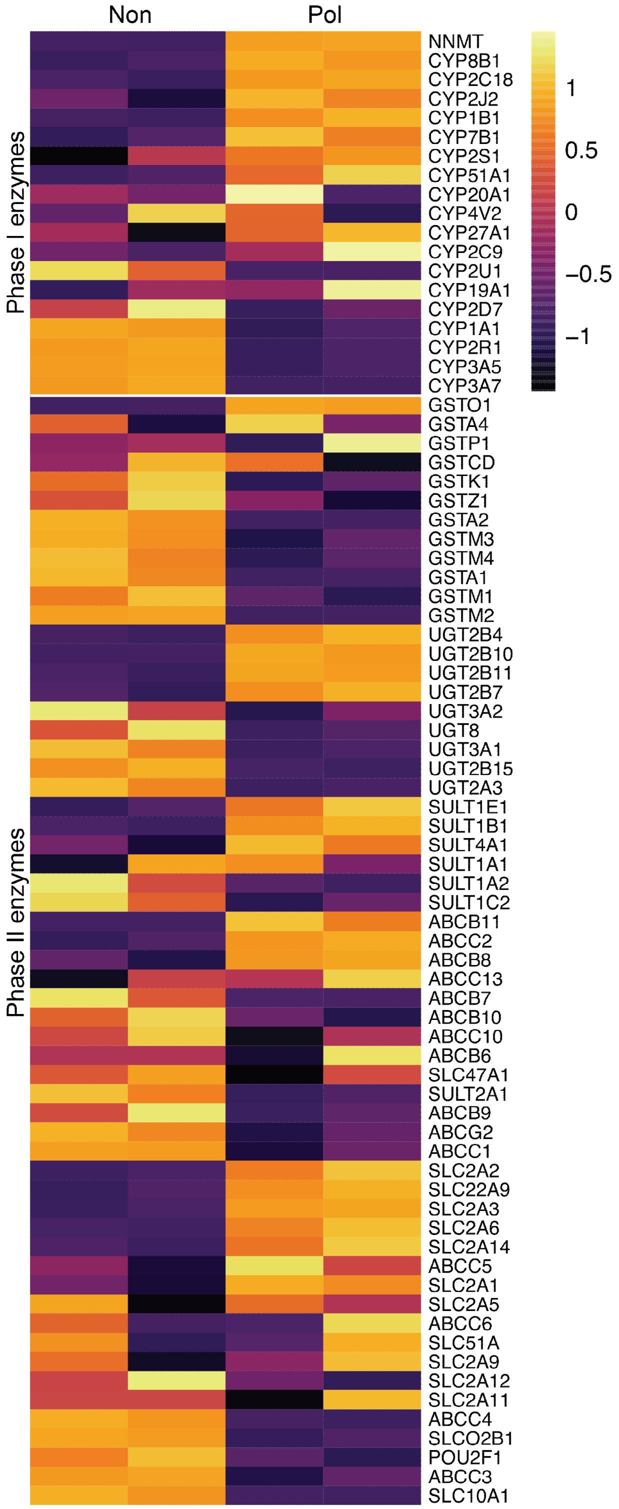

Supplementary Figure 9. Differential expression of drug-metabolizing enzymes. Heatmap of Z-score-normalized CPM values of phase I and II drug-metabolizing enzymes in nonpol- compared to pol-HLCs.

Supplemental Table 1

Gene	Forward primer (5'-3')	Reverse primer (3'-5')
RPS11	GCCGAGACTATCTGCACTAC	ATGTCCAGCCTCAGAACTTC
Nanog	TTTGTGGGCCTGAAGAAAACT	AGGGCTGTCCTGAATAAGCAG
CXCR4	ACTACACCGAGGAAATGGGCT	CCCACAATGCCAGTTAAGAAGA
KRT19	AACGGCGAGCTAGAGGTGA	GGATGGTCGTGTAGTAGTGGC
PROM1	AGTCGGAAACTGGCAGATAGC	GGTAGTGTTGTACTGGGCCAAT
AFP	TGGGACCCGAACTTTCCA	GGCCACATCCAGGACTAGTTTC
ALB	GGTGTTGATTGCCTTTGCTC	CCCTTCATCCCGAAGTTCAT
HEV	GGTGGTTTCTGGGGTGAC	AGGGGTTGGTTGGATGAA
APOB	TGCTCCACTCACTTTACCGTC	TAGCGTCCAGTGTGTACTGAC
APOC1	TCCAGTGCCTTGGATAAGCTG	GGCTGATGAGTTCCCGAGC
APOA2	CTGTGCTACTCCTCACCATCT	CTCTCCACACATGGCTCCTTT
MTTP	ACAAGCTCACGTACTCCACTG	TCCTCCATAGTAAGGCCACATC
HMGCR	TGATTGACCTTTCCAGAGCAAG	CTAAAATTGCCATTCCACGAGC
NPC1L1	AGAGTGAGCCTTACACAACCA	GCAGGACACGTTGGAGAGT
ABCA1	ACATCCTGAAGCCAATCCTGA	CTCCTGTCGCATGTCACTCC
SCARB1	ACTTCTGGCATTCCGATCAGT	ACGAAGCGATAGGTGGGGAT
CYP8B1	CTTGTTCGGCTACACGAAGGA	GCAGGGAGTAGACAAACCTTG
NR1H4	TGCAGATCAGACCGTGAATGA	TTGGTTGCCATTTCCGTCAAA
ABCB11	TTGGCTGATGTTTGTGGGAAG	CCAAAAATGAGTAGCACGCCT
ABCC2	TCTCTCGATACTCTGTGGCAC	CTGGAATCCGTAGGAGATGAAGA
P-gp	GGGATGGTCAGTGTTGATGGA	GCTATCGTGGTGGCAAACAATA
ABCG2	ACGAACGGATTAACAGGGTCA	CTCCAGACACACCACGGAT
SLC47A1	TCAACCAGGGAATTGTACTGC	CAGAGCCTATCACCCCAAGA
SLC22A9	CCCTCAGCCAAGATGCACTC	GCGTCACTTGTGTTGGGGAA
SLC51A	ACCTCGTTTTATGCCGTGTG	AAGAAGGCGTATTGGAAAGGG
SLC10A1	AAGGACAAGGTGCCCTATAAAGG	TTGAGGACGATCCCTATGGTG
SLCO1A2	TCAGCGATGAGCAGTTTCATT	TGGAATCCATTAAAGCGCCAA
ABCC3	CACCAACTCAGTCAAACGTGC	GCAAGACCATGAAAGCGACTC
NNMT	GAGATCGTCGTCACTGACTACT	CACACACATAGGTCACCACTG
CYP1B1	ACGTACCGGCCACTATCACT	CTCCCCACGACCTGATCCA
GSTO1	GAACGGCTGGAAGCAATGAAG	TGCCATCCACAGTTTCAGTTT
GSTM1	TCTGCCCTACTTGATTGATGGG	TCCACACGAATCTTCTCCTCT
UGT2B4	CAAATGTTGAGTTCGTTGGAGGA	CTGACGTGTTACTGACCATCG
UGT2B10	GAAATGGACTACAGTTCTGCTGA	GTGGATGAGTCGTTGGGATCA
CYP3A7	TGCTTTGTCCTTCCGTAAGGG	CAGCATAGGCTGTTGACAGTC
CYP3A4	AAGTCGCCTCGAAGATACACA	AAGGAGAGAACACTGCTCGTG
HAV	GGTAGGCTACGGGTGAAAC	AACAACTCACCAATATCCGC
