# Supplementary material for: Stem cell-derived polarized hepatocytes
Source: Nat Commun. 2020 Apr 3;11:1677. doi: 10.1038/s41467-020-15337-2 (PMC7125181; doi:10.1038/s41467-020-15337-2)
Supplement: Supplementary file 2 — Reporting summary [file 41467_2020_15337_MOESM2_ESM.pdf]

natureresearch

Corresponding author(s): Viet Loan Dao Thi, Xianfang Wu, Charles M Rice

Reporting Summary

Nature Research wishes to improve the reproducibility of the work that we publish. This form provides structure for consistency and transparency in reporting. For further information on Nature Research policies, see Authors & Referees and the Editorial Policy Checklist.

Statistical parameters

When statistical analyses are reported, confirm that the following items are present in the relevant location (e.g. figure legend, table legend, main text, or Methods section).

n/a	Confirmed	
☐	☒	The exact sample size (*n*) for each experimental group/condition, given as a discrete number and unit of measurement
☐	☒	An indication of whether measurements were taken from distinct samples or whether the same sample was measured repeatedly
☐	☒	The statistical test(s) used AND whether they are one- or two-sided<br>*Only common tests should be described solely by name; describe more complex techniques in the Methods section.*
☐	☒	A description of all covariates tested
☐	☒	A description of any assumptions or corrections, such as tests of normality and adjustment for multiple comparisons
☐	☒	A full description of the statistics including central tendency (e.g. means) or other basic estimates (e.g. regression coefficient) AND variation (e.g. standard deviation) or associated estimates of uncertainty (e.g. confidence intervals)
☐	☒	For null hypothesis testing, the test statistic (e.g. *F*, *t*, *r*) with confidence intervals, effect sizes, degrees of freedom and *P* value noted<br>*Give P values as exact values whenever suitable.*
☒	☐	For Bayesian analysis, information on the choice of priors and Markov chain Monte Carlo settings
☒	☐	For hierarchical and complex designs, identification of the appropriate level for tests and full reporting of outcomes
☒	☐	Estimates of effect sizes (e.g. Cohen's *d*, Pearson's *r*), indicating how they were calculated
☐	☒	Clearly defined error bars<br>*State explicitly what error bars represent (e.g. SD, SE, CI)*

Our web collection on statistics for biologists may be useful.

Software and code

Policy information about availability of computer code

Data collection	NIS-Elements BR version 4.10.01 was used for image acquisition.
Data analysis	Graphpad PRISM version 5.0 was used for statistical analysis. Fiji version 2.0.0-rc-69/1.52p was used for image analysis. RNA-seq data analysis was performed using open source software. Specifically, Seqtk version 1.2 and fastx_toolkit version 0.0.14 were used to process sequences in FASTQ format. Sequencing reads were aligned to the reference genome using TopHat2 version 2.0.12 with Bowtie version 2.2.7. Aligned reads were counted using featureCounts from subread version 1.4.6. Statistical analyses were performed using the edgeR Bioconductor package version 3.12.1 in the statistical computing environment R (www.r-project.org).

For manuscripts utilizing custom algorithms or software that are central to the research but not yet described in published literature, software must be made available to editors/reviewers upon request. We strongly encourage code deposition in a community repository (e.g. GitHub). See the Nature Research guidelines for submitting code & software for further information.

April 2018

Data

Policy information about availability of data

All manuscripts must include a data availability statement. This statement should provide the following information, where applicable:

- Accession codes, unique identifiers, or web links for publicly available datasets
- A list of figures that have associated raw data
- A description of any restrictions on data availability

Data sets have been deposited in NCBI's Gene Expression Omnibus and are accessible through GEO Series accession number GSE123462 (at https://www.ncbi.nlm.nih.gov/geo/query/acc.cgi?acc=GSE123462; private until publication).

Field-specific reporting

Please select the best fit for your research. If you are not sure, read the appropriate sections before making your selection.

☒ Life sciences ☐ Behavioural & social sciences ☐ Ecological, evolutionary & environmental sciences

For a reference copy of the document with all sections, see nature.com/authors/policies/ReportingSummary-flat.pdf

Life sciences study design

All studies must disclose on these points even when the disclosure is negative.

Sample size	Depending on the assay, we chose a sufficient number of biological replicates to produce data with errors at acceptably low level while avoiding making the study unnecessarily large, meaning high costs (for stem cell differentiation, RNA-seq analysis, and mass-spec analysis), logistical issues (sending biological samples to Singapore on dry ice while preserving the quality of the samples), and ethical issues (use of primary human hepatocytes).
Data exclusions	No data were excluded from the analyses.
Replication	Experiments revealing differences in measured data were repeated 3 - 4 times independently, as indicated in figure legends. For the RNA-seq analysis performed in Figure 2 and Supplemental Figure 8, experiments (including RNA extraction, library preparation, and next generation sequencing) were repeated independently twice. All results were successfully replicated.
Randomization	The study includes no experiments dependent on allocation of samples/organisms/participants into experimental groups.
Blinding	The study includes no experiments dependent on group allocation and blinding is thus not relevant.

Reporting for specific materials, systems and methods

Materials & experimental systems

n/a	Involved in the study
☒	☐ Unique biological materials
☐	☒ Antibodies
☐	☒ Eukaryotic cell lines
☒	☐ Palaeontology
☒	☐ Animals and other organisms
☒	☐ Human research participants

Methods

n/a	Involved in the study
☒	☐ ChIP-seq
☒	☐ Flow cytometry
☒	☐ MRI-based neuroimaging

Antibodies

Antibodies used	anti-FoxA2 (Cell Signaling, Cat#8186P), anti-HNF4α (Cell Signaling, Cat#3113S), anti-AFP (Sigma-Aldrich, St. Louis, MO, Cat#A8452), anti-ALB (Cedarlane, Burlington, Canada, Cat#CL2513A), anti-ZO1 (Thermo Fisher, Cat#402200), anti-E cadherin (Cell Signaling, Cat#3195), anti-SR-BI (Novus Biologicals, Cat#NB400-104), anti-BCRP (Millipore, CAT#MAB4155), anti-MRP2 (Cell Signaling, Cat#4446), anti-CYPB8B1 (Abcam, Cat# 129845), anti-ApoCIII (Abcam, Cat#AB21032), anti-ORF2 (a kind gift from Suzanne U. Emerson, NIH) and anti-HAV capsid (a kind gift from Stanley M. Lemon, UNC School of Medicine).
Validation	Anti-FoxA2, anti-HNF4α, anti-AFP, anti-ALB, anti-ZO1, anti-E cadherin, anti-SR-BI, anti-BCRP, anti-MRP2, anti-ApoCIII, anti-ORF2

and anti-HAV capsid antibodies were validated by immunfluorescence staining.
Anti-CYP8B1, anti-CYP8B1, and anti-ApoCIII were validated by western blot analysis.

Eukaryotic cell lines

Policy information about cell lines

Cell line source(s)	HUES8-iCas9 cells (Gonzalez et al. 2014, Cell Stem Cell; ExPASy Accession No CVCL_VR10)were a kind gift from Danwei Huangfu, MSKCC<br>H9 (WA09, hPSCreg Name WAe009-A) cells were purchased from WiCell<br>S10-3 cells (Emerson et al. 2010, JVI) were a kind gift from Suzanne Emerson at NIH
Authentication	Widely used human pluripotent stem cells, including WA09 and HUES8 were obtained from WiCell and Huangfu's laboratory at MSKCC, respectively. These cells were routinely tested for expression levels of relevant pluripotent and cellular factors under in study (for example they express pluripotent markers, APOC3 knockout cells were null for APOC3, and CYP8B1 knockdown cells displayed reduced levels of CYP8B1 upon hepatic differentiation) by western and/or DNA/RNA analyses. For stability, seed stocks of all pluripotent cell lines were frozen at early passages (P25 for H9 and P18 for HUES8). Experiments were performed using cells within 10 passages after recovery from liquid nitrogen. For sterility, pluripotent cell lines were tested routinely for mycoplasma. For phenotyping, the expression of pluripotent biomarkers such as POU5F1, NANOG, SSEA4, etc were routinely checked against a baseline description of the cell line, using gene expression analysis. For pluripotency, pluripotent cells were tested routinely for trilineage differentiation using qRT-PCR for germ layer marker expression.<br><br>S10-3 cells were subcloned from human hepatoma cell Huh7 and selected for their high efficiency to support hepatitis E virus replicaton. For mutation profile analysis, we performed amplicon sequencing and verified the mutation in KDR gene (1416A>T, Gln472His) in the S10-3 cells used in this study. For the stability, S10-3 cells have been expanded at early passages and frozen down using a seed lot system. Experiments were performed from the working lot, using cells within 5 passages after being recovered from liquid nitrogen. For sterility, S10-3 cells have been tested routinely for mycoplasma contamination. S10-3 cells have been routinely tested for expression levels of relevant factors (positive for CD24, CD133 and EpCAM, and negative for THY1) under this study by western and/or DNA/RNA analyses against a baseline description of the Huh7 cell line as reported previously (Hum Cell. 2018; 31(3): 261–267).
Mycoplasma contamination	All cell lines tested negative for mycoplasm contamination
Commonly misidentified lines<br>(See ICLAC register)	No commonly misidentified lines were used
